# The Predictors of Long COVID in Southeastern Italy

**DOI:** 10.3390/jcm12196303

**Published:** 2023-09-29

**Authors:** Vitaliano Nicola Quaranta, Andrea Portacci, Silvano Dragonieri, Cristian Locorotondo, Enrico Buonamico, Fabrizio Diaferia, Ilaria Iorillo, Sara Quaranta, Giovanna Elisiana Carpagnano

**Affiliations:** Department of Basic Medical Sciences, Neuroscience and Sense Organs, Section of Respiratory Disease, University “Aldo Moro” of Bari, 70121 Bari, Italy; vitalianonicola.40@gmail.com (V.N.Q.); a.portacci01@gmail.com (A.P.); cr.locorotondo@gmail.com (C.L.); enricobuonamico@gmail.com (E.B.); fabrizio.diaferia@gmail.com (F.D.); iorillo.ilaria@gmail.com (I.I.); sara.quaranta.02@gmail.com (S.Q.); elisiana.carpagnano@uniba.it (G.E.C.)

**Keywords:** Long COVID, COVID-19, pneumonia

## Abstract

Introduction: Long COVID is now recognized as a common consequence of the SARS-CoV-2 infection, but we are still far from fully understanding its pathogenesis and predictive factors. Many pathophysiological factors have been studied, including ethnicity. To our knowledge, the risk factors for Long COVID have not been studied in Southeastern Italy. Aims: The aim of this study was to evaluate the predictive factors of Long COVID in a cohort of patients from Southeastern Italy. Methods: We conducted a retrospective longitudinal study, enrolling inpatients and outpatients diagnosed with COVID-19 from June 2021 to March 2022. A total of 436 subjects were evaluated in an outpatient setting 12 weeks after a SARS-CoV-2 infection, recording comorbidities, symptoms, therapy, and clinical information. Univariate and multivariate binomial logistic regression analyses were performed on different risk factors to define the probability of developing Long COVID. Results: A total of 71.8% of patients (313) developed Long COVID, while the remaining 123 (28.3%) had a complete remission of symptoms 3 months after acute infection. During the acute phase of COVID-19, 68.3% of patients experienced respiratory failure and 81.4% received corticosteroid therapy. In a multivariate analysis, the female sex (SEX M ODD 0.513) and corticosteroids (ODD 2.25) were maintained as predictive values. Conclusions: From our data and in line with other studies, the female sex emerges as a risk factor for Long COVID in the population of Southeastern Italy. Corticosteroid therapy administered in the acute phase also appears to be associated with an increased risk of Long COVID. Although indications for the prescription of corticosteroid therapy in the acute phase were indicated by the presence of pneumonia complicated by respiratory insufficiency, there was an over-prescription of corticosteroid therapy in the real life of our cohort, with 64% of patients having respiratory insufficiency and 81% having corticosteroid therapy. We hypothesize that a synergistic link between viral infection and the side effects of corticosteroid therapy may arise in selected cases.

## 1. Introduction

The global challenge presented by the SARS-CoV-2 pandemic has resulted in millions of deaths and hundreds of millions of infections worldwide. However, it is believed that the actual number of cases is significantly higher, with the World Health Organization (WHO) estimating an underreporting rate of at least 50% [1]. COVID-19, although primarily a respiratory disease, can affect various organs, leading to a diverse range of symptoms associated with SARS-CoV-2 infection. These symptoms include neurological issues like loss of smell, headaches, muscle problems, and loss of taste, as well as respiratory problems such as cough, sore throat, and difficulty breathing. Gastrointestinal symptoms like diarrhea and vomiting as well as musculoskeletal symptoms like muscle pain can also be experienced [2].

Some individuals continue to experience these symptoms for more than 12 weeks or develop them after the acute phase of COVID-19, which is known as Long COVID [3]. Long COVID has had a significant epidemiological impact, affecting an estimated 65 million people globally [4]. The reasons why symptoms persist beyond three weeks in a variable percentage of patients ranging from 10% to 35% and up to 75% are still unknown [5,6,7]. The lack of knowledge about the post-COVID-19 syndrome can be attributed, in part, to the diverse population groups studied [5,6,7].

The pathophysiology of post-viral syndromes appears to be a complex interaction between viral loads and the immune response of the host [8]. This interaction can result in both direct non-immunologic effects and indirect immune effects [9]. Various hypotheses have been proposed, including the persistence of inflammatory reactions [10], the presence of lingering SARS-CoV-2 in tissues [11], muscle deconditioning [3], or post-traumatic stress disorder [3]. Furthermore, it is worth noting that the symptoms of Long COVID bear a striking resemblance to well-documented phenomena observed in other diseases, such as HIV-1, herpes simplex virus 1, and the Ebola virus disease. While there are differences in the cells where these viruses reside and their targeting mechanisms, several viruses share similar neuropsychiatric effects on the host. The most commonly reported comorbidities in these infections include anxiety, depression, and post-traumatic stress, likely attributable to the viruses’ capacity to invade the central nervous system and their ability to “hide” within CNS-resident structures, which contributes to latent infections and persistent disease [12]. Additionally, the reported prolonged pathological post-viral condition may overlap or have close associations with various diseases, including myalgic encephalitis/chronic fatigue syndrome (ME/CFS). ME/CFS is a debilitating clinical condition characterized by unexplained and persistent post-exertional fatigue, accompanied by an array of symptoms related to cognitive, immunological, endocrinological, and autonomic dysfunction, as assessed by Sukocheva et al. [13].

Although one-third of the cases do not exhibit conditions associated with Long COVID, several risk factors have been identified, including female sex, type II diabetes mellitus, and connective tissue disorders [4]. According to the European Respiratory Society, the risk factors for Long COVID are not necessarily linked to the initial severity of the illness but instead to female sex, age, and the number of symptoms experienced at onset [14].

Long COVID is a heterogeneous and multifactorial syndrome that can also be influenced by ethnicity [15], and it can manifest with a wide range of symptoms [14]. Currently, no predictive studies on Long COVID have specifically been conducted on a cohort of patients from Southeastern Italy. Therefore, the objective of this study is to assess the predictive factors of Long COVID within a cohort of patients from Southeastern Italy.

## 2. Materials and Methods

A total of 436 consecutive adult patients, both inpatients and outpatients, who had been diagnosed with COVID-19 between June 2021 and March 2022 at the Respiratory Disease Department of Policlinico in Bari, Italy, were enrolled in this study. Twelve weeks after their SARS-CoV-2 infection, all participants were evaluated at the outpatient clinic. The patients were recruited either after being discharged from the Respiratory Disease Department of Policlinico (55.7%) or through the Post COVID care pathway established by the Puglia region without ever being hospitalized (44.3%).

The inclusion criteria for the study were age over 18 years, COVID-19 infection occurring at least 12 weeks prior to the outpatient visit, and negativity for SARS-CoV-2 at the time of the visit, confirmed through molecular swab testing. Exclusion criteria included the presence of mental illnesses and/or non-compliance that hindered proper medical history taking.

This study was approved by the Institutional Review Board of Policlinico of Bari (Ethical Committee number: 6380; Approval date: 12 May 2020). The procedures used in this study adhere to the tenets of the Declaration of Helsinki.

The study design was retrospective and longitudinal in nature. A diagnosis of Long COVID was established if patients exhibited symptoms related to COVID-19 persisting for more than 12 weeks, with no alternative explanations for the symptoms and following the COVID-19 rapid guideline.

For each patient, the following pre-COVID-19 infection data were collected (Table 1): sex, age, body mass index (BMI), smoking status, SARS-CoV-2 vaccination history (including the number of doses received), and previous COVID-19 infection. Comorbidities were assessed using the Charlson index [16]. The presence of the following comorbidities was also recorded: diabetes mellitus (DM), arterial hypertension (AH), cardiovascular disease (CVD), chronic obstructive pulmonary disease (COPD), intestinal lung disease (ILD), other pulmonary diseases, dyslipidemia, cerebrovascular disease, dementia, autoimmune disease (AD), immunosuppression, history of cancer, current cancer, and thyroid disease.

The study defined two time periods: T0 represented the acute phase of COVID-19, and T1 referred to 3 months after the COVID-19 infection. Data related to the COVID-19 infection period were recorded as follows (Table 1):

- COVID-19 + duration: the number of days between the positive and negative results of the nasal molecular swab for COVID-19.
- Home Care/Hospitalization: indicating whether patients received care at home or were hospitalized.
- ICU Hospitalization: indicating whether patients required admission to the Intensive Care Unit.
- The presence or absence of Acute Respiratory Failure (ARF).
- Length of Hospitalization: recorded as 0 days for patients who were never hospitalized.
- The intensity of respiratory support: categorized as Ambient Air (AA) and Spontaneous Breath, Oxygen Therapy (OT), Non-Invasive Ventilation (NIV) or Continuous Positive Airway Pressure (CPAP), or Invasive Mechanical Ventilation (IMV) via Endotracheal intubation (ETI), or tracheostomy.
- We recorded antibiotic or antiviral treatments administered in our cohort, which included macrolides, β-lactams, fluoroquinolones, remdesivir, nirmatrelvir/ritonavir, convalescent plasma therapy, and monoclonal antibody therapy.
- The presence or absence of a cycle of corticosteroid therapy at a dosage of 6 mg/kg of dexamethasone or equivalent dosages of other corticosteroids in accordance with guidelines from the World Health Organization (WHO) [17] and the Infectious Diseases Society of America (ISDA) [18]. In hospitalized patients, corticosteroid therapy during the acute phase of COVID lasted for a minimum of 10 days. However, the precise duration remains unknown. Additionally, data on the duration and starting day of corticosteroid administration were not recorded.
- Administration of Low Molecular Weight Heparin (LMWH) based on medical indication.

Radiological diagnostics were not performed for all patients receiving home care. Among the 436 enrolled patients, 165 underwent Computed Tomography (CT) scans, while 287 underwent Radiography (X-ray). The presence of pneumonia and its unilateral or bilateral location, as well as the presence of pulmonary embolism (PE), pneumothorax (PNX), or pleural effusion (PlE) were recorded.

**Figure 1:**
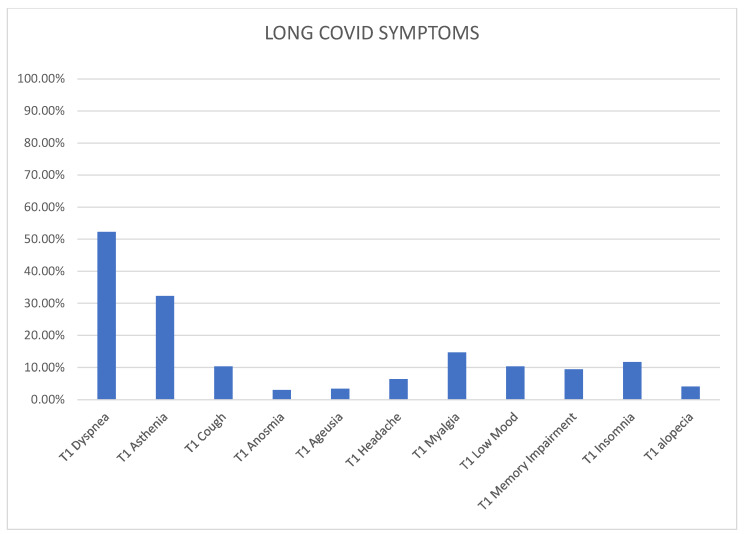
The distribution of Long COVID symptoms.

Symptoms reported by the patients at T0-time (acute phase) and T1-time (3 months after infection) were documented (Table 1 and Figure 1). Additionally, information regarding therapy administered during the acute phase of COVID-19, such as macrolide therapy and corticosteroid therapy following recommended guidelines from the ISDA [18] and WHO [17] in terms of dosage and duration were obtained, and the use of Low Molecular Weight Heparin (LMWH) were recorded.

*Statistical analysis* was performed to assess the distribution of data using the Kolmogorov–Smirnov test. Continuous variables with a non-parametric distribution were expressed as medians with interquartile ranges (25–75th percentiles). Categorical values were analyzed using the chi-square test or Fisher’s exact test when appropriate and reported as counts and percentages. The Mann–Whitney U test was used to compare non-normally distributed continuous variables. Univariate binomial logistic regression analyses were conducted to determine the probability of having the Long COVID disease. We verified the accuracy of the multivariate models using the Area Under the Curve (AUC). A significance level of *p* < 0.05 was considered statistically significant. All statistical analyses were performed using SPSS for Windows 25.0 software (SPSS, Chicago, IL, USA).

## 3. Results

We included a total of 436 patients in our study, with 43.6% being female. The median age of the participants was 58 years, and their average BMI was 28. Among the patients, 55.7% had been hospitalized for COVID-19 treatment, while 44.3% had received care at home. Remdesivir, nirmatrelvir/ritonavir, convalescent plasma, and monoclonal antibody therapy were administered only to hospitalized patients. The remaining therapies, specifically macrolides, β-lactams, and fluoroquinolone antibiotics, were prescribed both at home and in hospital settings.

After 3 months of COVID-19 infection and subsequent negative molecular swabs, 71.8% of the patients (313 individuals) experienced persistent symptoms, indicating Long COVID. The remaining 28.3% (123 patients) showed a complete resolution of their symptoms (Table 1). None of the patients were receiving chronic corticosteroid therapy at that moment. Among the 313 Long COVID patients, 264 (84.3%) had undergone a course of corticosteroid therapy, following the recommended dosage of 6 mg/kg of dexamethasone or equivalent (e.g., methylprednisolone 32 mg or prednisolone 40 mg) according to the guidelines from the World Health Organization (WHO) [17] and the Infectious Diseases Society of America (ISDA) [18].

The median number of total symptoms among the Long COVID patients was 1 (ranging from 0 to 2). Graph number 1 illustrates the most commonly reported symptoms at T1-time among those who developed Long COVID. Dyspnea was the most prevalent symptom, affecting 52.3% of patients, followed by asthenia (32.3%), myalgia (14.7%), insomnia (11.7%), low mood (10.3%), memory impairment (9.4%), headache (6.4%), cough (10.3%), alopecia (4.1%), anosmia (3.0%), and ageusia (3.4%).

### 3.1. Comparison between Groups

#### 3.1.1. Comparison Based on Different Levels of Respiratory Support

As can be seen from Supplementary Table S1, we categorized the patients into four subgroups based on their respiratory support:

Ambient Air (AA) and Spontaneous Breath (*n* = 136);

Oxygen Therapy (OT) (*n* = 190);

Non-Invasive Ventilation (NIV)/Continuous Positive Airway Pressure (CPAP) (*n* = 74);

Endotracheal Intubation (ETI)/Tracheotomy (Tracheo) (*n* = 34).

Among these subgroups, it is notable that the patients requiring Endotracheal Intubation (ETI) or Tracheotomy (Tracheo) had a lower percentage of females (26.5%) compared to, for instance, the AA subgroup (52.9%). Furthermore, the ETI/Tracheal subgroup exhibited the lowest percentage of COPD (8.8%), the highest percentage of dyslipidemia (55.9%), and a higher percentage of dyspnea (64.7%), cough (100%), and asthenia.

Patients in the AA and Spontaneous Breath subgroup, on the other hand, displayed the highest frequency of nausea (10.9%) and anosmia (47.1%). When considering the two extremes of severity (AA vs. ETI/Tracheal), the more critically ill patients were more likely to have bilateral pneumonia (ETI/Tracheal: 82.4%; AA: 42.6% without pneumonia) and pulmonary embolism (ETI/Tracheal: 17.6%; AA: 0%). They also had a higher likelihood of receiving corticosteroid therapy (ETI/Tracheal: 97.1%; AA: 61.6%), LMWH (ETI/Tracheal: 94.1%; AA: 19.6%), macrolide therapy (ETI/Tracheo: 68.8%; AA: 94.1%), β-lactam antibiotics therapy (ETI/Tracheo: 88.2%; AA: 15.9%), and Convalescent Plasma therapy (ETI/Tracheo: 11.8%; AA: 0.7%).

#### 3.1.2. Comparison between the Long COVID and Recovered COVID-19 Patients

**Figure 2:**
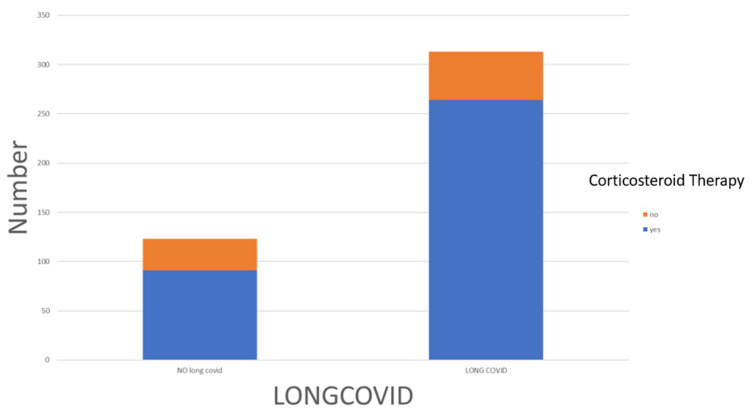
The distribution of corticosteroid therapy among Long COVID and no-Long COVID patients.

Comparison between the Long COVID patients (pt) and the control group (Recovered COVID-19 pt) revealed some differences. As shown in Table 1, Long COVID patients (*n* = 313) had a higher percentage of females compared to the control group (*n* = 123) consisting of asymptomatic patients (46.6% vs. 35.8%; *p* = 0.025), as well as a higher incidence of hospitalization (58.5% vs. 48.8%). During the acute phase of COVID-19 (T0-time), patients who later developed Long COVID had a higher incidence of dyspnea (60.1% vs. 47.2%), asthenia (85.0% vs. 77.2%), and headache (26.2% vs. 17.9%). Long COVID patients during the acute phase were also more likely to have received oral or intravenous corticosteroid therapy (84.3% vs. 74%; *p* = 0.010) (Figure 1). However, other parameters examined in the study, such as hospitalization rate (58.5% vs. 48.8%) or ICU admission (15% vs. 12.2%), did not show significant differences between Long COVID patients and the control group (Figure 2).

We compared Long COVID with Recovered COVID-19 within the subgroups of home care and hospitalization, respectively, with regard to the therapy administered during the acute phase. The outcomes of these analyses have been summarized in Supplementary Table S2.

The significance of corticosteroid therapy is preserved when analyzing the population in subgroups based on the treatment setting (see Supplementary Table S2). In both the Home Care subgroup (*n* = 193; 65.4% vs. 50.8%; *p* = 0.037) and the hospitalized patient subgroup (97.8% vs. 91.7%; *p* = 0.043), the percentage of patients receiving corticosteroid therapy during the acute phase of COVID-19 was significantly higher.

### 3.2. The Predictors of Long COVID

In terms of predicting Long COVID, our univariate binomial logistic regression analysis identified several factors. Female sex (odds ratio for male sex = 0.637 (0.414–0.980); *p* = 0.040), higher BMI (odds ratio = 1.051 (1.005–1.098); *p* = 0.028), the presence of dyspnea at T0-time (odds ratio = 1.686 (1.107–2.566); *p* = 0.015), and the administration of corticosteroid therapy at T0-time (odds ratio = 1.895 (1.143–3.140); *p* = 0.013) were identified as predictive factors for Long COVID (see Table 2).

After conducting a multivariate analysis, only two parameters remained statistically significant. Female sex (odds ratio for male sex = 0.513 (0.316–0.833); *p* = 0.007) and corticosteroid therapy (odds ratio = 2.255 (1.285–3.956); *p* = 0.005) retained their significance as predictive factors for Long COVID (see Table 2).

As can be seen in Supplementary Table S3, we verified the accuracy of the multivariate logistic models on the prediction of Long COVID by calculating the Area Under the Curve (AUC). The initial model, incorporating the significant parameters identified in the univariate analysis (sex, BMI, dyspnea, and corticosteroid therapy), yielded an AUC of 0.646 (0.586–0.705). Recognizing the clinical significance of the treatment setting parameter (home care or hospital care), a second model was constructed, and its accuracy with an AUC of 0.645 (0.585–0.706) was comparable to the first model.

## 4. Discussion

Our research identified the factors that can predict the occurrence of Long COVID in a group of 436 patients from Southeast Italy. Consistent with previous studies [19,20], we found that female sex in our population is a risk factor for Long COVID, as determined through multivariate analysis (odds ratio for male sex = 0.513 (0.316–0.833); *p* = 0.007). Additionally, we observed for the first time that receiving corticosteroid therapy orally or intravenously during the acute phase of COVID-19 appears to increase the risk of experiencing at least one symptom related to COVID-19 persisting for at least 3 months after infection (odds ratio = 2.255 (1.285–3.956); *p* = 0.005).

From the subgroup analysis based on respiratory support in our population, there is an observable trend where the proportion of individuals identifying as female declines as the severity of respiratory compromise increases (e.g., AA = 52.9% vs. OT = 75.3% vs. NIV/CPAP = 63.5% vs. ETI/TRACHEO = 26.5%; *p* = 0.020). This finding aligns with the existing literature, which consistently indicates that being female is associated with a protective effect against ICU admission (Odds Ratio: 0.259 [95% Confidence Interval: 0.089–0.921]; *p* = 0.036) [21].

Despite similar prevalence between men and women, men with COVID-19 face a higher risk of experiencing unfavorable outcomes and mortality, irrespective of age [22]. Although there is no direct evidence establishing testosterone as the primary cause of increased susceptibility in men, it is conceivable that testosterone plays a role in promoting ACE-2 and TMPRSS2 expression, potentially facilitating viral entry and fusion [23].

In line with the recent findings outlined in a recent review [24], our data shows that a greater BMI is associated with an increased requirement for respiratory support.

In our research sample, we noted a reduced incidence of COPD in patients undergoing AA and spontaneous breathing (9.4%) in contrast to 20% in those receiving different levels of respiratory support (OT: 20%; NIV/CPAP: 20.3%; ETI/TRACHEO: 3.5%). This finding aligns with observations made in previous patient groups, where the significance of COPD as a risk factor for severe COVID-19 was underscored [25,26].

Similarly, we observed how patients undergoing ETI/tracheo had a higher percentage of CVD, a data consistent with what is described in the literature [27].

Overall, 71.8% of our population developed Long COVID, which is a rate similar to other studies. For example, in a cohort of 1655 hospitalized COVID patients, 76% reported the persistence of at least one symptom (primarily fatigue or muscle weakness) after 6 months [6]. In contrast to most studies that focused on post-discharge hospitalized patients or outpatients, our study included both categories of patients, representing a wider range of clinical severity [28]. The incidence rates of Long COVID varied among different patient cohorts due to the heterogeneity of the enrolled population and the multifactorial nature of Long COVID‘s pathogenesis [4]. Initially, Long COVID was described in approximately 10% of patients [5], while the persistence of symptoms after three weeks in mild COVID-19 cases managed on an outpatient basis ranged from 10% to 35% [7]. The wide array of Long COVID symptoms has necessitated a comprehensive approach to the condition. Between February and May 2022, a national online survey involving 124 centers focused on long-term COVID care was conducted. The majority of these centers adopted a multifaceted, interdisciplinary approach for both diagnosing and treating long-term COVID. Among these centers, approximately half had specialized support in cardiology, respiratory diseases, radiology, infectious diseases, neurology, and psychology [29].

Ethnicity appears to influence symptom manifestation, with dyspnea and asthenia being the most prevalent symptoms in Southeastern Italy, as well as in other European countries. In Asian populations, however, studies have reported a lower prevalence of fatigue and dyspnea at a follow-up of 3–6 months [28].

Our study revealed that female sex is an independent risk factor for the persistence of COVID-related symptoms for more than 3 months. This finding aligns with the results of previous studies conducted on different populations, indicating the consistent association between female sex and Long COVID [30,31], although some studies have not found this association [32], highlighting the heterogeneity of the Long COVID condition [14]. The previous observations of SARS survivors showed higher levels of depression and anxiety in females [33]. Some authors have also reported a greater predisposition for women to develop fatigue or shortness of breath [19]. The underlying mechanism for these consequences may involve the viral infection’s direct effects, an immunological response, or corticosteroid therapy [20].

Among the 313 patients with Long COVID, 264 (84.3%) had received a course of corticosteroid therapy at the recommended dosage of 6 mg/kg of dexamethasone or equivalent (e.g., methylprednisolone 32 mg or prednisolone 40 mg) for severe and critical COVID-19 cases [17,18]. Unfortunately, we did not record information on the duration and timing of corticosteroid administration during the acute phase, which may have influenced our study’s results. However, the significant association between corticosteroid therapy and the persistence of Long COVID symptoms, demonstrated in both univariate and multivariate analyses, suggests a potential link between the therapy and Long COVID, despite the absence of this information.

The role of corticosteroid therapy in viral infections has always been a subject of debate. While it is indicated during the acute phase of severe and critical COVID-19 to control cytokine release syndrome (CRS) in advanced stages [17,34], numerous scientific articles caution against routinely using corticosteroids for viral pneumonia treatment [34,35]. In viral pneumonia, corticosteroid administration can increase viral replication, weaken host defenses, and elevate the risk of secondary infections [34]. Likewise, it may hinder the adaptive immune response and promote viral replication in the early stages or mild forms of COVID-19 [17,18]. In our real-life retrospective study, we observed the overprescription of corticosteroid therapy in 88 mild COVID patients without respiratory insufficiency, likely due to the fear of COVID-19’s unfavorable progression, limited hospital resources, scarce treatment options, and conflicting information. Consequently, corticosteroid prescription was favored over a wait-and-observe approach. One possible explanation for our findings is that in some patients, the administration of corticosteroid therapy during the acute phase—particularly outside the recommended guidelines—could contribute to the persistence of the virus in non-respiratory system reservoirs. COVID-19 has tropism in virtually all systems, and even with negative nasopharyngeal swabs, viremic phases could occur over time, increasing the risk of symptom persistence. However, it is worth noting that in a selected subgroup of 49 patients with abnormal computed tomography (CT) findings and resting hypoxia or exertional desaturation, systemic steroid therapy (deflazacort) for 8–10 weeks led to clinical improvement. These authors emphasized the risk of immunosuppression in this specific subgroup [36]. Therefore, further studies are needed to fully understand the role of corticosteroids in COVID and Long COVID, and precision medicine approaches may be necessary in their administration, considering factors such as patient characteristics, dosage, and therapy. Another limitation of this study is the lack of information during an extended follow-up period.

## 5. Conclusions

In summary, our study aims to shed light on the potential connection between corticosteroid therapy administered during the acute phase of COVID-19 and the development of Long COVID. However, the absence of essential information, including the duration and timing of therapy, impedes us from drawing a conclusive assessment. Future studies should further explore this association, taking into account the duration and timing of corticosteroid therapy administration in order to validate or refute our findings. Precision medicine approaches will be crucial in determining the appropriate use of corticosteroids during the acute phase of COVID-19, considering individual patient characteristics and optimizing therapy.

## Figures and Tables

**Table 1:** The General Population and a Comparison between Long COVID vs. no Long COVID patients.

	General Population (*n* = 436)	Long COVID-19 Pt (*n* = 313)	Recovered COVID-19 Pt (*n* = 123)	*p* Value
Sex F n (%)	190 (43.6)	146 (46.6)	44 (35.8)	0.025 *
Age (y) IQ (25–75)	58 (50.25–66.00)	58 (51–66)	59 (49 66.25)	n.s.
BMI Kg/m^2^ IQ (25–75)	28 (25–31)	28 (25–31.75)	27 (24–30)	n.s.
Smoke n (%) No Ex yes	225 (51.8) 167 (38.5) 42 (9.7)	159 (51.0) 128 (41.0) 25 (8.0)	66 (54.1) 39 (32.0) 17 (13.9)	n.s.
Charlson Index IQ (25–75)	2 (1–3)	2 (1–3)	2 (1–3)	n.s.
DM n (%)	82 (18.8)	62 (19.8)	20 (16.3)	n.s.
AH n (%)	210 (48.2)	157 (50.2)	53 (43.1)	n.s.
CVD n (%)	85 (19.5)	63 (20.1)	22 (17.9)	n.s.
COPD n (%)	74 (17.0)	57 (18.2)	17 (13.8)	n.s.
ILD n (%)	13 (3.0)	7 (2.2)	6 (4.9)	n.s.
Other Pulmonary Disease n (%)	39 (8.9)	28 (8.9)	11 (8.9)	n.s.
Cerebrovascular Disease n (%)	21 (4.8)	17 (5.4)	4 (3.3)	n.s.
Dyslipidemia n (%)	144 (33.0)	105 (33.5)	39 (31.7)	n.s.
Dementia n (%)	6 (1.4)	5 (1.6)	1 (0.8)	n.s.
AD n (%)	30 (6.9)	22 (7.0)	8 (6.5)	n.s.
Immunosuppression n (%)	2 (0.5)	2 (0.6)	1 (0.8)	n.s.
History of Cancer n (%)	21 (4.8)	15 (4.8)	6 (4.9)	n.s.
Current Cancer n (%)	15 (3.4)	10 (3.2)	5 (4.1)	n.s.
Home Care/Hospitalization n (%) Hospitalization Home Care	243 (55.7) 193 (44.3)	183 (58.5) 130 (41.5)	60 (48.8) 63 (51.2)	0.042 *
ICU yes n (%)	62 (14.2)	47 (15.0)	15 (12.2)	n.s.
ARF yes n (%)	298 (68.3)	216 (69.0)	82 (66.7)	n.s.
Hospitalization Length (day)	10 (0–24)	11 (0–25)	0 (0–20.25)	n.s.
Tot. Symptoms T0	1 (1–3)	1 (1–3)	0 (1–3)	n.s.
Dyspnea T0 yes n (%)	246 (56.4)	188 (60.1)	58 (47.2)	0.010 *
Cough n (%)	326 (74.8)	234 (74.8)	92 (74.8)	n.s.
Asthenia n (%)	361 (82.8)	266 (85.0)	95 (77.2)	n.s.
Nausea T0 yes n (%)	38 (8.7)	31 (9.9)	7 (5.7)	n.s.
Vomiting T0 yes n (%)	24 (5.5)	18 (5.8)	6 (4.9)	n.s.
Diarrhea T0 yes n (%)	70 (16.1)	50 (16.0)	20 (16.3)	n.s.
Headache T0 yes n (%)	104 (23.9)	82 (26.2)	22 (17.9)	0.042 *
Anosmia T0 yes n (%)	158 (36.2)	115 (36.7)	43 (35.0)	n.s.
Ageusia T0 yes n (%)	152 (34.9)	111 (35.5)	41 (33.3)	n.s.
Corticosteroid Therapy yes n (%)	355 (81.4)	264 (84.3)	91 (74.0)	0.010 *
LMWH yes n (%)	267 (61.2)	199 (63.6)	68 (55.3)	n.s.
Macrolide Therapy Yes n (%)	359 (82.5)	260 (83.3)	99 (80.5)	n.s.
β-lactam antibiotics therapy	186 (42.7)	138 (44.1)	48 (39.1)	n.s.
Fluoroquinolones therapy	28 (6.8)	22 (7.3)	6 (5.5)	n.s.
Remdesivir therapy	24 (5.5)	18 (5.8)	6 (4.9)	n.s.
Nirmatrelvir/ritonavir therapy	14 (3.2)	10 (3.2)	4 (3.3)	n.s.
Convalescent Plasma therapy	15 (3.4)	12 (3.8)	3 (2.4)	n.s.
Monoclonal Antibody therapy	3 (0.7)	3 (1.0)	1 (0.8)	n.s.
Respiratory Support n (%) AA and Spontaneous Breath OT NIV/CPAP ETI/tracheo	138 (31.7) 190 (43.6) 74 (17.0) 34 (7.8)	97 (31.0) 143 (45.7) 47 (15.0) 26 (8.3)	41 (33.3) 47 (38.2) 27 (22) 8 (6.5)	n.s.
# Pneumonia Localization n (%) No Pneumonia Monalateral Bilateral	19 (6.6) 81 (28.2) 187 (65.2)	14 (6.5) 66 (30.6) 136 (63.0)	5 (7.0) 15 (21.1) 51 (71.8)	n.s.
# Pnx yes n (%)	5 (1.7)	3 (1.4)	2 (2.8)	n.s.
## PlE n (%)	18 (6.3)	12 (5.6)	6 (8.5)	n.s.
PE yes n (%)	17 (10.3)	14 (11.7)	3 (6.7)	n.s.
COVID19 + duration day IQ (25–75)	23.5 (17–32)	24 (17–32)	23 (18–32)	n.s.
Reinfection n (%)	31 (7.1)	21 (6.7)	10 (8.1)	n.s.
Number Vaccine doses IQ (25–75)	1 (0–1)	1 (1–2)	1 (1–1)	n.s.

Abbreviations: IQ 25–75 = Interquartile 25–75%; Pt = patients; y = years; BMI = Body Mass Index; Kg/m^2^ = Kilogram/meter^2^; DM = diabetes mellitus; AH = arterial hypertension; CVD = cardiovascular disease; COPD = chronic obstructive pulmonary disease; ILD = intestinal lung disease; AD = autoimmune disease; ICU = Intensive Care Unit; ARF = Acute Respiratory Failure Yes; TOT Symptoms T0 = number of symptoms at T0 time; LMWH = Low Molecular Weight Heparin; AA = Ambient Air; OT = Oxygen Therapy; NIV = Non Invasive Ventilation; CPAP = Continuous Positive Airway Pressure; ETI = Endotracheal intubation; Tracheo = tracheotomy; PNX = Pneumothorax; PlE = Pleural Effusion; PE = Pulmonary Embolism; n.s. = not significant. # = 149 missing value; ## = 271 missing value; *: *p* value < 0.050.

**Table 2:** A Prediction of Long COVID: Univariate and Multivariate Binary Logistic Regression Analysis.

	Univariate Logistic Regression			Multivariate Logistic Regression		
	ODD	CI 95%	*p* Value	ODD	CI 95%	*p* Value
Sex M	0.637	0.414–0.980	0.040 *	0.513	0.316–0.833	0.007 *
Age (y)	1.000	0.984–1.016	n.s.			
BMI Kg/m^2^	1.051	1.005–1.098	0.028 *	1.047	0.999–1.097	0.056
Smoke yes	1.149	0.918–1.439	n.s.			
Charlson Index	1.059	0.946–1.187	n.s.			
DM yes	1.272	0.731–2.213	n.s.			
AH yes	1.329	0.873–2.023	n.s.			
CVD yes	1.157	0.676–1.980	n.s.			
COPD yes	1.388	0.772–2.497	n.s.			
ILD yes	0.446	0.147–1.355	n.s.			
Other Pulmonary Disease yes	1.0	0.482–2.078	n.s.			
Cerebrovascular Disease yes	1.709	0.563–5.183	n.s.			
Dyslipidemia yes	1.087	0.696–1.699	n.s.			
Dementia yes	1.981	0.229–17.126	n.s.			
AD yes	1.087	0.470 −2.511	n.s.			
Immunosuppression yes	0.785	0.070–8.732	n.s.			
History of Cancer yes	0.982	0.372–2.591	n.s.			
Current Cancer yes n (%)	0.585	0.230–1.490	n.s.			
Home Care/Hospitalization	0.677	0.445–1.029	0.068			
ICU yes	1.272	0.682–2.371	n.s.			
ARF yes	1.130	0.724–1.764	n.s.			
Hospitalization Length (day)	1.011	0.998–1.024	n.s.			
Tot. Symptoms T0	1.141	0.988–1.317	n.s.			
Dyspnea T0 yes	1.686	1.107–2.566	0.015 *	1.338	0.838–2.138	0.222
Cough	0.998	0.617–1.613	n.s.			
Asthenia	1.609	0.949–2.728	n.s.			
Nausea T0 yes	1.822	0.780–4.254	n.s.			
Vomiting T0 yes	1.190	0.461–3.072	n.s.			
Diarrhea T0 yes	0.979	0.556–1.725	n.s.			
Headache T0 yes	1.630	0.964–2.756	n.s.			
Anosmia T0 yes n	1.081	0.699–1.671	n.s.			
Ageusia T0 yes	1.099	0.707–1.708	n.s.			
Corticosteroid Therapy yes	1.895	1.143–3.140	0.013 *	2.255	1.285–3.956	0.005 *
LMWH yes	1.412	0.924–2.156	n.s.			
Macrolide Therapy Yes	1.212	0.709–2.072	n.s.			
β-lactam antibiotics therapy yes	1.232	0.805–1.886	n.s.			
Fluoroquinolones therapy yes	1.349	0.532–3.420	n.s.			
Remdesivir therapy yes	1.190	0.461–3.072	n.s.			
Nirmatrelvir/ritonavir therapy yes	0.982	0.302–3.191	n.s.			
Convalescent Plasma therapy yes	1.595	0.442–5.751	n.s.			
Monoclonal Antibody therapy yes	1.181	0.122–11.460	n.s.			
Respiratory Support AA and Spontaneous Breath OT NIV/CPAP Tracheo/ETI	Ref 0.728 0.936 0.536	0.304–1.742 0.397–2.208 0.213–1.348	n.s.			
# Pneumonia Localization n (%) No Pneumonia Monalateral Bilateral	Ref 1.050 1.650	0.360–3.063 0.864–3.149	n.s.			
# Pnx yes	0.484	0.080–2.968	n.s.			
## PlE	0.637	0.230–1.765	n.s.			
PE yes	1.849	0.505–6.765	n.s.			
COVID19 + duration day	0.994	0.978–1.011	n.s.			
Reinfection	0.813	0.371–1.779	n.s.			
Number Vaccine doses	1.112	0.844–1.465	n.s.			

Abbreviation: CI 95 = Confidence Interval 95%; y = years; BMI = Body Mass Index; Kg/m^2^ = Kilogram/meter^2^; ICU = Intensive Care Unit; ARF = Acute Respiratory Failure Yes; TOT Symptoms T0 = number of symptoms at T0 time; LMWH = Low Molecular Weight Heparin; AA = Ambient Air; OT = Oxygen Therapy; NIV = Non Invasive Ventilation; CPAP = Continuous Positive Airway Pressure; ETI = Endotracheal intubation; Tracheo = tracheotomy; PNX = Pneumothorax; PlE = Pleural Effusion; PE = Pulmonary Embolism; DM = diabetes mellitus; AH = arterial hypertension; CVD = cardiovascular disease; COPD = chronic obstructive pulmonary disease; ILD = intestinal lung disease; AD = autoimmune disease. n.s. = not significant. # = 149 missing value; ## = 271 missing value; *: *p* value < 0.050.

## Data Availability

Not applicable.

## Supplementary Materials

The following supporting information can be downloaded at: https://www.mdpi.com/article/10.3390/jcm12196303/s1, Figure S1: Distribution of Long COVID symptoms; Table S1: Subgroup analysis: comparison based on different levels of respiratory support; Table S2: Comparison of Long COVID with Recovered COVID-19 within the subgroups of home care and hospitalization in terms of the therapy administered during the acute phase; Table S3: The Prediction of Long COVID.
