# Supplementary material for: The Predictors of Long COVID in Southeastern Italy
Source: J Clin Med. 2023 Sep 29;12(19):6303. doi: 10.3390/jcm12196303 (PMC10573494; doi:10.3390/jcm12196303)
Supplement: Supplementary file 1 [file jcm-12-06303-s001.zip › jcm-2602700-supplementary.pdf]

Table S1. Subgroup analysis: comparison based on different levels of respiratory support.

	AA and Spontaneous Breath (n=136)	OT (n=190)	NIV/CPAP (n=74)	ETI/tracheo (n=34)	P value
Long Covid	97 (70.3)	143 (75.3)	47 (63.5)	26 (76.5)	n.s.
Sex F n(%)	73 (52.9)	79 (41.6)	29 (39.2)	9 (26.5)	0.020*
Age (y) IQ (25 - 75)	54 (45.25 – 62.75)	60 (53.25 – 69)	60 (53.25 - 69)	54 (46 - 59)	
BMI Kg/m^2 IQ (25 - 75)	26 (23 - 30)	28 (26 - 32)	28 (24 – 30.5)	32 (28 - 34)	0.005*
Smoke n(%) yes	23 (16.8)	10 (5.3)	6 (8.1)	3 (8.8)	n.s.
Charlson Index IQ (25 - 75)	2 (0 - 3)	2 (1 - 4)	2 (1-3)	2 (1 -3)	
DM n(%)	23 (16.7)	31 (16.3)	21 (28.4)	7 (20.6)	n.s.
AH n(%)	61 (44.2)	93 (48.9)	38 (51.4)	18 (52.9)	n.s.
CVD n(%)	20 (14.5)	43 (22.6)	11 (14.9)	11 (32.4)	0.046*
COPD n(%)	13 (9.4)	38 (20.0)	15 (20.3)	8 (23.5)	0.038*
ILD n(%)	3 (2.2)	8 (4.2)	2 (2.7)	0 (0)	n.s.
Other Pulmonary Disease n(%)	12 (8.7)	14 (7.4)	6 (8.1)	7 (20.6)	n.s.
Cerebro Vascular Disease n(%)	8 (5.8)	9 (4.7)	4 (5.4)	0 (0)	n.s.
Dyslipidemia n(%)	32 (23.2)	64 (33.7)	32 (43.2)	16 (47.1)	0.00622*
Dementia n(%)	1 (0.7)	3 (1.6)	2 (2.7)	0 (0)	n.s.
AD n(%)	12 (8.7)	13 (6.8)	3 (4.1)	2 (5.9)	n.s.
Immunosuppression n(%)	0 (0)	1 (0.5)	1 (1.4)	1 (2.9)	n.s.
Hystory of Cancer n(%)	3 (2.2)	14 (7.4)	3 (4.1)	1 (2.9)	n.s.
Current Cancer n(%)	3 (2.1)	8 (4.2)	5 (6.8)	0 (0)	n.s.
Dyspnoea T0 yes n(%)	68 (49.3)	121 (63.7)	35 (47.3)	22 (64.7)	0.015*
Cough n (%)	31 (22.5)	190 (100)	71 (95.9)	34 (100)	0.000*
Asthenia n (%)	65 (47.4)	190 (100)	72 (97.3)	34 (100)	0.000*
Nausea T0 yes n(%)	15 (10.9)	16 (8.4)	6 (8.1)	1 (2.9)	0.000*
Vomiting T0 yes n(%)	8 (5.8)	9 (4.7)	5 (6.8)	2 (5.9)	n.s.
Diarrhea T0 yes n(%)	21 (15.2)	31 (16.3)	13 (17.6)	5 (14.7)	n.s.
Headache T0 yes n(%)	39 (28.3)	38 (20.0)	21 (28.4)	6 (17.6)	n.s.
Anosmia T0 yes n(%)	65 (47.1)	57 (30.0)	25 (33.8)	11 (32.4)	0.014*
Ageusia T0 yes n(%)	59 (42.8)	59 (31.1)	25 (33.8)	9 (26.5)	n.s.
Corthicosteroid Therapy yes n(%)	85 (61.6)	176 (92.6)	57 (77.0)	33 (97.1)	0.000*
LMWH yes n(%)	27 (19.6)	162 (85.3)	46 (62.2)	32 (94.1)	0.000*
Macrolide Therapy Yes n(%)	95 (68.8)	169 (88.9)	63 (85.1)	32 (94.1)	0.000*
β-lactam antibiotics therapy	22 (15.9)	106 (55.8)	28 (37.8)	30 (88.2)	0.000*
Fluoroquinolones therapy	13 (11.0)	11 (5.8)	3 (4.3)	1 (2.9)	n.s.

Remdesivir therapy	2 (1.4)	16 (8.4)	4 (5.4)	2 (5.9)	n.s.
Paxlovid therapy	1 (0.7)	7 (3.7)	4 (5.4)	2 (5.9)	n.s.
Convalescent Plasma therapy	1 (0.7)	7 (3.7)	3 (4.1)	4 (11.8)	0.004*
Monoclonal Antibody therapy	0 (0.0)	2 (1.1)	2 (2.7)	0 (0)	n.s.
# Pneumonia Localization n(%)<br>No Pneumonia<br>Monalateral<br>Bilateral	 <br>18 (42.6)<br>6 (15.4)<br>15 (38.5)	 <br>0 (0)<br>54 (31.4)<br>118 (68.6)	 <br>1 (2.4)<br>15 (37.5)<br>26 (61.9)	 <br>0 (0)<br>6 (17.6)<br>28 (82.4)	0.000*
# Pnx yes n(%)	0 (0)	4 (2.3)	1 (2.4)	0 (0)	n.s.
## PlE n(%)	0 (0)	8 (4.7)	4 (9.5)	6 (17.6)	0.009*
PE yes n(%)	0 (0)	11 (10.8)	2 (10.0)	4 (16.7)	n.s.
COVID19+ duration day<br>IQ (25 - 75)	21.5 (16 - 30)	25 (17 -32)	22 (16 - 32)	27 (22.5 - 38)	
Reinfection n(%)	13 (9.4)	12 (6.3)	3 (4.1)	3 (8.8)	n.s.
Number Vaccine doses IQ (25 - 75)	1 (0 - 1)	0 (0 - 1)	1 (0 - 1)	0 (0 - 1)	n.s.

Abbreviations: IQ 25-75= Interquartile 25 – 75%; Pt= patients; y=years; BMI= Body Mass Index; Kg/m^2= $Kilogram/meter^2$; DM=diabetes mellitus arterial: AH= arterial hypertension; CVD= cardiovascular disease; COPD=chronic obstructive pulmonary disease; ILD= intestinal lung disease; AD= autoimmune disease; ICU=Intensitive Care Unit; ARF=Acure Respiratory Failure Yes; TOT Symptoms T0= number of symptoms at T0 time; LMWH= Low Molecular Weight Heparin; AA= Ambient Air; OT= Oxygen Therapy; NIV= Non Invasive Ventilation; CPAP=Continuous Positive Airway Pressure; ETI=Endotracheal intubation; Tracheo= tracheotomy; PNX= Pneumothorax; PlE= Pleural Effusion; PE=Pulmonary Embolism; n.s.= not significant.

#= 149 missing value; ##=271 missing value

*: p value< 0.050

Table S2. Comparison Long Covid to Recovered Covid-19 within the subgroups of home care and hospitalization in terms of the therapy administered during the acute phase.

	HOME CARE GROUP n =193			HOSPITALIZATION GROUP		
	Long Covid-19 pt (n=130)	Recovered Covid-19 pt (n=63)	P value	Long Covid-19 pt (n=)	Recovered Covid-19 pt (n=)	P value
Corthicosteroid Therapy yes n(%)	85 (65.4)	32 (50.8)	0.037	179 (97.8)	55 (91.7)	0.043*
LMWH yes n(%)	32 (24.6)	12 (19.0)	n.s.	167 (91.3)	56 (93.3)	n.s.
Macrolide Therapy Yes n(%)	88 (67.7)	44 (67.7)	n.s.	172 (94.0)	57 (95.0)	n.s.
β-lactam antibiotics therapy	10 (7.7)	6 (9.5)	n.s.	128 (69.9)	42 (70)	n.s.
Fluoroquinolones therapy	17 (14.3)	5 (10.2)	n.s.	5 (2.7)	1 (1.7)	n.s.
Remdesivir therapy #				18 (9.8)	6 (10.0)	n.s.
Nirmatrelvir/ritonavir therapy #				10 (5.5)	4 (6.7)	n.s.
Convalescent Plasma therapy #				12 (6.6)	3 (5.0)	n.s.
Monoclonal Antibody therapy #				3 (1.6)	0 (0)	n.s.

Abbreviation:LMWH= Low Molecular Weight Heparin
n.s.= not significant
# No home prescriptions
*: p value< 0.050

Table S3. Prediction of Long Covid

	Univariate Logistic Regression			Multivariate Model 1				Multivariate Model 2			
	ODD	CI 95%	P value	ODD	CI 95%	P value	AUC	ODD	CI 95%	P value	AUC
Sex M	0.637	0.414 – 0.980	0.040*	0.513	0.316 – 0.833	0.007*	0.646 (0.586 – 0.705)	0.507	0.311 – 0.827	0.006	0.645 (0.585 – 0.706)
Age (y)	1.000	0.984 – 1.016	0.976								
BMI Kg/m^2	1.051	1.005 – 1.098	0.028*	1.047	0.999 – 1.097	0.056		1.046	0.998 – 1.096	0.063	
Home Care/Hospitalization	0.677	0.445 – 1.029	0.068					0.910	0.539 – 1.536	0.724	
Dyspnea T0 yes	1.686	1.107 – 2.566	0.015 *	1.338	0.838 – 2.138	0.222		1.333	0.834 – 2.130	0.229	
Corticosteroid Therapy yes	1.895	1.143 – 3.140	0.013*	2.255	1.285 – 3.956	0.005*		2.151	1.159 – 3.993	0.015	

Abbreviation:
BMI= Body Mass Index
n.s.= not significant
# No home prescriptions
*: p value< 0.050
